# Risk factors for mental health symptoms during the COVID-19 pandemic in ophthalmic personnel and students in USA (& Canada): a cross-sectional survey study

**DOI:** 10.1186/s12888-021-03535-1

**Published:** 2021-10-26

**Authors:** Yi Pang, Meng Li, Connor Robbs, Jingyun Wang, Samiksha F. Jain, Ben Ticho, Katherine Green, Donny Suh

**Affiliations:** 1 Illinois College of Optometry, Chicago, IL USA; 2 Department of Health and Behavioral Sciences, University of Colorado Denver, Denver, CO USA; 3 State University of New York College of Optometry, New York, NY USA; 4 University of Nebraska Medical Center, Omaha, NE USA; 5 Ticho’s Eye Association, Chicago, IL USA; 6 NOVA Southeastern University, Fort Lauderdale, FL USA

**Keywords:** Mental health, COVID-19, Depression, Anxiety, Stress, Ophthalmologist, Optometrist

## Abstract

**Background:**

The COVID-19 pandemic poses mental health challenges to frontline healthcare workers. Eye care professionals may be especially susceptible to mental health problems due to high-risk exposures to patients. Yet, no prior research has studied mental health issues among eye care professionals during the COVID-19 pandemic.

**Objective:**

The purpose of this study was to identify risk factors for mental health problems during the COVID-19 pandemic among eye care professionals.

**Methods:**

We conducted a cross-sectional survey study among eye care professionals and students in the United States and Canada from June 23 to July 8, 2020 during the COVID-19 pandemic. A total of 8505 eye care professionals and students received email invitations to the survey and 2134 participated. We measured mental health outcomes including symptoms of depression, anxiety, and stress using validated scales, as well as potential risk factors including demographic characteristics, state-level COVID-19 case counts, participants’ patient interactions, childcare responsibilities, and pre-pandemic stress levels. Linear multiple regression and logistic regression analyses were used to determine relationships between risk factors and mental health outcomes.

**Results:**

We found that 38.4% of eyecare professional participants in the survey met screening threshold as probable cases of anxiety, depression, or both during the COVID-19 pandemic. Controlling for self-reported pre-pandemic stress level and state COVID-19 case daily cases, significant risk factors for depression, anxiety, and psychological stress during the COVID-19 pandemic included: being female, younger age, and being Black or Asian. Interestingly, we found two somewhat surprising protective factors against depression symptoms: more frequent interactions with patients and having a greater proportion of childcare responsibilities at home.

**Conclusions:**

This study showed a high prevalence of mental health problems and revealed disparities in mental health among eye care personnel and students: Female, younger, Black, and Asian populations are particularly vulnerable to mental health issues. These results indicate that it is critical to identify mental health issues more effectively and develop interventions among this population to address this significant and growing public health issue. The strategies and policies should be reflective of the demographic disparities in this vulnerable population.

**Supplementary Information:**

The online version contains supplementary material available at 10.1186/s12888-021-03535-1.

## Introduction

During the COVID-19 pandemic, frontline healthcare workers are at higher risk for mental health disorders because they face increased infection risk, a heavier workload, and potentially, life or death decisions [1–6]. At the beginning of the COVID-19 pandemic, the WHO recognized the high burden of COVID-19 pandemic on healthcare workers and called for action to address the immediate needs to protect healthcare workers from risks of infection [7]. However, it is equally important to protect healthcare workers from risks of mental health problems. The severity of healthcare workers’ mental health problems is illustrated clearly in a meta-analysis on 65 studies, which showed that the prevalence of anxiety, depression, and stress were 31.8, 40.3, and 11.4% among healthcare workers during COVID-19 [2].

Besides emergency healthcare workers, eye care professionals may be one of the groups of highest COVID-19 infection risk due to physical proximity to patients’ nose and mouth during eye examinations. Thus, eye care professionals may be especially at risk for stress and mental health issues. However, no existing literature has studied the mental health impacts of COVID-19 on eyecare professionals as a targeted population [3, 5, 8–11]. The current study fills this gap and examines risk factors for mental health issues among eyecare professionals during the COVID-19 pandemic.

The challenges specific to eye care professionals during the COVID-19 pandemic are dictated by the position of the eyes, a short distance from the nose and mouth, with physical connection between the ocular surface and the nasopharynx via the nasolacrimal duct. Because of the short inter-personal distance with patients, major components of eye examination, such as slit lamp biomicroscopy, retinoscopy, indirect and direct ophthalmoscopy, and contact lens manipulation, can pose increased infection risk for eye care professionals. Furthermore, eye care professionals have to deal with a series of ocular manifestations associated with COVID-19, such as conjunctivitis, a symptom frequently reported in patients with COVID-19 [12, 13]. It is not surprising that the first doctor who reported COVID-19, Dr. Wenliang Li, was an ophthalmologist in China, who died due to professional contacts with COVID-19 patients. Protection against infection is often lacking among eye care professionals. Besides regular use of personal protective equipment, eye care professionals had to be creative to protect themselves in examinations unique to the discipline, such as creating a shield over the slit lamps and phoropter. All of these factors can impose extra stress on eye care professionals during the COVID-19 pandemic, with the potential to cause mental health issues among this population.

The pandemic related mental health problems are not only impacting professional healthcare workers, but potentially also impacting prospective healthcare professionals in training. Mental health difficulties are already alarmingly prevalent among college students, particularly in the United States, with every eight in ten students experiencing frequent stress episodes in 2019 [14]. The top contributors to mental health difficulties in college students have been reported as pressure to succeed, educational performance, and post-college graduation plans [15]. These challenges are expected to be similar, and perhaps even greater among students in professional schools. Negative impacts of COVID-19 on mental health have been reported in both college students [16, 17] and health care students [18, 19]. However, no studies have specifically reported the mental health impacts of the COVID-19 pandemic among professional eye-care students. The current research filles this gap by including eyecare students in our study of mental health impacts of the COVID-19 pandemic.

In order to protect eyecare professionals and students from mental health problems such as depression, anxiety, and psychological stress during the COVID-19 pandemic, it is important to identify factors associated with the risk for these mental health issues among this population. For example, what demographic sectors within eyecare professionals are at greater risk for mental health issues? What life-situations act as risk factors or protective factors for mental health issues among this population? Understanding these questions can help inform the design of targeted interventions for mental health problems in the eyecare profession.

The goal of the current study was to identify risk factors for mental health issues during the COVID-19 pandemic among eyecare professionals. We utilized a cross-sectional survey among eyecare doctors, staff, and students in the U.S. and Canada to answer this question. We included validated scales to measure depression, anxiety, and psychological stress as outcomes in the survey, and included demographic measures, professional contact with patients, as well as childcare responsibilities as potential predictors. We also recorded self-reported pre-pandemic stress level, and state level COVID-19 cases during the pandemic as control variables.

## Methods

The study was a cross-sectional online survey including 23 questions. Original Survey questions can be found in the Supplemental Document. The research protocol and informed consent forms were approved by the Institutional Review Board (IRB) of the Illinois College of Optometry (Chicago, IL) with IRB number of 19,032. Adherence to the Health Insurance Portability and Accountability Act (HIPAA) was maintained during this study.

### Recruitment method

We sent an email invitation for an online survey using Survey Monkey platform (https://www.surveymonkey.com) on June 23, 2020 to 8505 eye care professionals (Ophthalmologists and Optometrists), staff, and optometry students in 7 U.S. institutions and one professional organization, including: the Department of Ophthalmology at University of Nebraska Medical Center Omaha, members of Pediatric Ophthalmology Listservs, State University of New York College of Optometry, Pennsylvania College of Optometry at Salus University, Illinois College of Optometry, New England College of Optometry, Southern California College of Optometry at Marshall B. Ketchum University, and College of Optometry at Nova Southeastern University. We did not employ random sampling to achieve a representative sample. Instead, we recruited a convenience sample of eyecare professional and students by contacting eyecare institutions via the first author’s professional connections, and did our best to select institutions from a wide range of geographic regions. Our professional connections at these institutions then distributed the survey invitation to professionals and students within each institution. Initial email recipients received a reminder email on July 5th, 2020, and data collection ended on July 8, 2020. During this period, the total confirmed cases of COVID-19 passed the 3,000,000 mark in the United States [20]. A total of 2134 respondents completed the survey.

### Survey questions

#### Predictor measures

The survey collected demographic information, including gender, age range, race, ethnicity, occupation (type of position within the eyecare profession), geographic location (state in the USA, or in Canada). In addition, participants reported the proportion of childcare responsibility they were responsible for, since childcare responsibilities may add additional psychological strain to participants’ lives. Participants also reported their patient interactions in two questions: how long they had been involved in patient care since March 15, 2020 (roughly when the pandemic lockdown procedures started in the U.S.), and how frequently they were involved in patient care in the past 2 weeks (number of days/week). Participants’ names were not collected. Survey ID was used to assign subject number. There was no time limit for participants to finish questionnaire and participants could review the previous questions. IP address of the participant’s computer was not used to track entry. Cookie was used to assign a unique user identifier to each participant. Permission to use cookies was asked on the first page of the survey. Cookie was valid for 90 days for the survey. 

#### Mental health measures

This study focused on symptoms of depression, anxiety, and psychological stress as outcome variables. We used the 2-item depression scale (PHQ-2) and 2-item anxiety scale (GAD-2) of the validated Patient Health Quesionnaire-4 [21] to measure depression and anxiety symptoms, respectively. We used the 4-item short form of the validated Perceived Stress Scale (PSS-4) to measure psychological stress [22, 23]. Scoring in each scale followed established protocol [21–23]: Each question in the depression (PHQ-2) and anxiety (GAD-2) scales was scored on a 0–3 scale: not at all = 0, several days = 1, more than half the days = 2, and nearly every day = 3, with the final score calculated as the sum of scores from the two items, ranging from 0 to 6. The threshold for detecting probable cases of depression and anxiety in each scale was a score greater than or equal to 3 [21]. Each question in the psychological stress scale (PSS-4) was recorded on a 5-point scale from 0 to 4: never = 0, almost never = 1, sometimes = 2, fairly often = 3, very often = 4, and reverse coded for question items 2 & 3, and the mean score across items constitute the final score for psychological stress [22, 23]. There was no threshold for identifying probable cases of stress because stress is not a clinical diagnosis.

In addition to the three main outcome measurements, we also administered a simple 1-item question on self-reported stress before the COVID-19 pandemic and during the pandemic (the past 2 weeks), respectively. Because we had no historical data on the psychological wellbeing of participants prior to the pandemic, the one-item self-report measure of pre-pandemic stress serves as a crude assessment of participants’ general psychological wellbeing at baseline, providing a crude comparison of stress before vs. during the pandemic. Lastly, participants were asked what aspects of life had positively or negatively impacted their mental health, as well as what activities helped them maintain mental health, which are not included in the analysis since they are not the focus of the study.

### State level COVID-19 case data

We retrieved the state-level total COVID-19 case data from the July 8th, 2020 update on Our World in Data [20], the state-level averaged new daily COVID-19 case data across 3 weeks from June 21–July 8, 2020 updates on NPR [24], and computed total cases and daily cases per million population using the 2001 state population from the U.S. Census Bureau [25].

### Sample size calculation

The following formula was used to calculate sample size: n = (z)^2^ p (1-p) / d^2^ with a 95% level of confidence (α =0.05, Z = 1.96), margin of error d = 5%, and proportion of expected mental health comorbidities *p* = 41% The proportion of doctors with mental health comorbidities was estimated at 41% based on a recent study by Torjesen [18]. To allow for subgroup analyses, the target sample size was amplified from 372 to 1000 participants. The eventual sample size exceeded this target due to a higher-than-expected response rate among email recipients.

### Data exclusion

We received a total of 2135 survey responses. One response from a participant was deleted because it contained only demographic information but no response to other survey questions. The survey platform used cookies to prevent multiple responses from the same computer, but we conducted additional procedures to safeguard against repeat participants: We asked participants to enter their email address for a chance to win a gift card, and 88% of responses provided an email address; we excluded 10 duplicate entries associated with an email address following an earlier survey entry from the same email address. After all exclusions, the final sample size was 2124.

### Statistical analysis

All data were analyzed using SPSS Statistics 27.0 software with *P* < 0.05 to determine the statistical significance. Descriptive statistics were applied. Percentage of participants with probable cases of depression and anxiety were calculated based on clinical threshold from the depression and anxiety scales (equal to or above 3 points on a 0–6 point scale), respectively. Multiple regression analyses were conducted to determine risk factors for depression, anxiety, and psychological stress scores during the pandemic, controlling for self-reported stress level prior to the pandemic as well as demographic factors (categorical variables were dummy coded as indicated in Tables 2 & 3), where the raw scores in the depression, anxiety, and stress scales were used as continuous outcome variables. Diagnostic tests were conducted to verify regression assumptions. We found all outcome variables to be largely normally distributed with normality and kurtosis smaller than or close to 1 with no apparent outliers. Mahalanobis distance was computed in all linear regressions to identify multi-variate outliers, and 10 potential outliers were identified (extreme Mahalanobis distance scores above 190 in all three regressions, when the rest of the cases had Mahalanobis distance all below 64 and falling within a normal distributions), and these 10 cases were the only respondents in the regression who reported gender identity as not male or female, which may have contributed to the large Mahalanobis distance; we conducted the linear regressions with and without these potential outliers and found similar results, and decided to retained these cases in the final analyses we present in this paper. Residue plots from linear regressions indicate good multivariate normality, homoscedasticity, and linearity. Multicollinearity diagnostics showed no sign of multicollinearity, with all condition index below 30.

A similar set of two logistic regressions were also conducted using the same set of predictors as the linear regressions, but dichotomous outcomes of whether the participants fit the criterion for potential cases of depression and anxiety, respectively.

### Data availability

The dataset supporting the conclusions of this article is available in the Open Science Framework repository at https://osf.io/6qfru/.

## Results

We received 2124 unique responses from participants. This represents a response rate of 25.0% out of the original 8505 email recipients. Of the 2124 participants, 29.9% were male and 68.5% female, 0.2% non-binary, 0.1% trans non-binary, 0.1% identity not listed, 0.4% declined to specify, and 0.8% missed gender information; median age was in the 30–39 age bracket; the overwhelming majority of participants resided in 50 USA states (92.7%), with some residing in Canada (1.8%) and 5.5% did not report location of residence; job positions included 41.6% optometrists, 11.6% ophthalmologists, 37.4% optometry students, and 8.7% eye care staff (0.8% had missing occupation information). The demographic characteristics of the participants are listed in Table 1.

**Table 1:** Demographic characteristics of the participants (*n* = 2124)

Characteristic	N (%)
**Gender**	
Female	1455 (68.5)
Male	636 (29.9)
Non-binary	5 (0.2)
Trans non-binary	2 (0.1)
Identity not listed	2 (0.1)
Decline to specify	8 (0.4)
Missing	16 (0.8)
**Race**	
White	1321 (62.2)
Black	87 (4.1)
Asian	538 (25.3)
American Indian/Alaska native	2 (0.1)
Native Hawaiian or Other Pacific Islander	9 (0.4)
More than one race	68 (3.2)
Decline to specify	86 (4.0)
Missing	13 (0.6)
**Ethnicity**	
Hispanic/Latino	138 (6.5)
Non-Hispanic/Latino	1811 (85.3)
Decline to specify	159 (7.5)
Missing	16 (0.8)
**Age (years)**	
20–29	896 (42.2)
30–39	409 (19.3)
40–49	284 (13.4)
50–59	264 (12.4)
60–69	195 (9.2)
70–79	58 (2.7)
80+	10 (0.5)
Missing	8 (0.4)
**Job categories**	
Ophthalmologist	267 (11.6)
Optomologist	833 (41.6)
Staff	184 (8.7)
Student	794 (37.4)
Missing	16 (0.8)
**Location**	
United States	1969 (92.7)
Canada	39 (1.8)
Missing	16 (5.5)

### Self-reported stress before vs. during the COVID-19 pandemic

On the 1-item self-reported stress measurement (a scale of 1–5), participants reported significantly higher stress during the pandemic compared to before, a level of 3.49 (*SD* = 1.12) vs. 2.86 (*SD* = 1.02), *t* (2111) = 23.18, *P* < 0.001, Cohen’s D = 1.25. This crude comparison nonetheless paints a clear picture of the additional stress COVID-19 brought on among our participants.

### Scores on depression, anxiety, and psychological scale

The depression and anxiety screening questions showed 38.4% of all participants (*n* = 815) as probable cases of either depression, anxiety, or both: This includes 34.3% of participants scoring above the threshold as probable cases of anxiety (scoring ≥3 on a 0–6 point scale), 20.9% scoring above the threshold as probable cases of depression (scoring ≥3 on a 0–6 point scale), and 16.3% scoring above the threshold for both. The 4-item scale on psychological stress showed a mean of 1.54 (SD = 0.75) on a 0–4 point scale.

Three separate multiple regressions were conducted on participants’ scores in the depression screening, anxiety screening, and psychological stress scale, respectively. To account for mental health burdens from different life-circumstances before the COVID-19 pandemic, all regressions used the single-item self-reported stress level pre-COVID-19 as a control variable. Other predictors included: age, gender (dummy coded, with male as reference category), race/ethnicity (dummy coded, with non-Hispanic whites as reference category), occupation (dummy coded, with optometrists as reference category), proportion of childcare responsibilities at home, number of weeks the participant had patient interactions since March 15, 2020, number of days/week the participant had patient interactions in the past 2 weeks, total COVID-19 cases per million population in their state of residence (as of July 8, 2020) [12], mean daily new cases per million population in their state of residence (June 21–July 8, 2020, averaged across 3 weeks of data) [16].

Table 2 lists the standardized coefficients for all predictors in the three regressions on depression, anxiety, and psychological stress, respectively. As expected, greater self-reported stress prior to the pandemic predicted higher scores on depression, anxiety, and psychological stress during the pandemic (*Ps* < 0.01). Controlling for pre-COVID-19 stress and other predictors, the following demographic factors significantly predicted the mental health outcomes. Younger participants experienced marginally higher levels of depression (*ß = -0.06, P* = 0.05), and significantly higher levels of anxiety (*ß = -0.13, P* < 0.01) and psychological stress (*ß = -0.13, P* < 0.01) during the COVID-19 pandemic; females experienced more anxiety and psychological stress during the pandemic compared to males (*ß = 0.11 and ß = 0.10, respectively, Ps* < .01 for both); Black and Asian participants showed more symptoms of depression (*ß* = 0.06, *P* = .01, and *ß* = 0.09, *P* < .01, respectively) and psychological stress (*ß* = 0.07, *P* < .01, and *ß* = 0.06, *P* = .01, respectively) compared to non-Hispanic Whites. In addition, optometry students experienced greater levels of depression (*ß* = 0.08, *P* = .03) and psychological stress (*ß* = 0.10, *P* = .01) compared to optometrists, whereas ophthalmologists and staff largely did not differ from optometrists mental health symptoms, except ophthalmologists experienced marginally fewer anxiety symptoms than optometrists (*ß* = -0.05, *P* = .05). Controlling for all other predictors, we also found a surprising effect of childcare responsibility and patient interactions on depression: Greater childcare responsibilities and more days/week with patient interactions both predicted lower levels of depression (*ß* = − 0.07, *P* < .01, and *ß* = − 0.10, *P* = .01, respectively). State-level case numbers only showed one significant effect: average daily new cases during data collection predicted greater psychological stress (*ß* = 0.05, *P* = .05), however total cases had no effect on any of the three outcome variables.

**Table 2:** Multiple Regressions Predicting Depression Score, Anxiety Score, and Psychological Stress Score

	Outcome Variable					
Predictor	Depression Score (*n* = 1772)		Anxiety Score (*n* = 1776)		Psychological Stress (*n* = 1782)	
	*ß*	*P*	*ß*	*P*	*ß*	*P*
Stress before Covid-19	0.18	<.01	0.24	<.01	0.22	<.01
Age	-0.06	.05	-0.13	<.01	-0.13	<.01
**Gender (ref: male)**^**a**^						
Female	0.01	.78	0.11	<.01	0.10	<.01
Other gender	0.01	.55	0.05	.04	0.02	.49
**Race (ref: non-Hispanic white)**^**a**^						
Black	0.06	.01	0.03	.15	0.07	<.01
Asian	0.09	<.01	0.01	.73	0.06	.01
Hispanic	0.01	.54	0.02	.46	0.02	.37
Other race/ethnicity	0.03	.17	0.04	.11	0.04	.09
**Job position (ref: optometrists)**^**a**^						
Ophthalmologists	−0.02	.50	−0.05	.05	0.01	.60
Staff	0.03	.19	0.01	.67	0.01	.57
Students	0.08	.03	0.05	.18	0.10	.01
Childcare responsibility	−0.07	<.01	−0.03	.18	−0.03	.22
Daily new cases (per thousand)	0.03	.18	0.03	.23	0.05	.05
Total cases (per thousand)	−0.03	.18	− 0.01	.72	0.00	.90
Patient interaction-weeks since 3/15/2020	0.06	.18	0.03	.45	−0.02	.62
Patient interaction-days/week (past 2 weeks)	−0.10	.01	−0.01	.83	−0.05	.19
Model Adjusted R^2^	0.10		0.14		0.19	

**Notes:** ^a^ Gender, race, and job position were all dummy coded with the reference group noted in parentheses

We also analyzed depression and anxiety as dichotomous outcomes in logistic regressions, based on whether the depression or anxiety score was above the criterion in the screening scale to be identified as a potential case of depression or anxiety (equal to or above 3 on a scale of 0–6), respectively. These two logistic regressions used the same set of predictors as the multiple regressions described above. Note that psychological stress is not a screening tool with a clear-cut threshold for potential diagnosis, and therefore, could not be analyzed as a dichotomous outcome.

Table 3 lists the results from the logistic regressions. The findings showed similar patterns as those from the multiple regressions described above. Stress before COVID-19 was a significant predictor for the likelihood of depression (OR = 1.42) and anxiety (OR = 1.56), *P* < .01 for both. Younger participants had marginally greater odds for depression (OR = 0.88, *P* = .05) and a greater odd for anxiety (OR = 0.82, *P* < .01). Being female again was associated with greater odds for anxiety (OR = 1.70, P < .01), but in addition, different from the multiple regression results, self-identifying as other gender categories also predicted higher odds for anxiety (OR = 7.89, *P* = .02). As in linear multiple regressions, being Black and being Asian both predicted greater odds for depression (OR = 1.76, *P* = .048, and OR = 1.58, *P* < .01, respectively). Similar to results from multiple regressions, having a greater proportion of the childcare responsibilities at home, as well as more frequent interactions with patients per week both acted as protective factors against depression (OR = 0.81 and OR = 0.86, P < .01 for both). A result that wasn’t identified before, however, is that greater childcare responsibilities at home also protected participants against anxiety (OR = 0.87, *P* = .01). In the logistic regressions, we found a different pattern of results with regard to job position compared to the multiple regression results above: Being a student (compared to optometrists) did not impact the odds of depression; however, we did find that ophthalmologists had lower odds for anxiety compared to optometrists (OR = 0.50, P = .01), even though this factor was only a marginally significant predictor in the multiple regression analysis.

**Table 3:** Logistic Regressions Predicting Potential Case of Depression and Anxiety

	Outcome Variable					
Predictor	Depression (n = 1772)			Anxiety (n = 1776)		
	*OR*	*95% CI*	*P*	*OR*	*95% CI*	*P*
Stress before Covid-19	1.42	[1.26, 1.61]	<.01	1.56	[1.39, 1.74]	<.01
Age	0.88	[0.77, 1.00]	.05	0.82	[0.73, 0.92]	<.01
**Gender (ref: male)**^**a**^						
Female	1.14	[0.85, 1.53]	.37	1.70	[1.31, 2.2]	<.01
Other gender	3.02	[0.72, 12.60]	.13	7.89	[1.45, 42.99]	.02
**Race (ref: non-Hispanic white)**^**a**^						
Black	1.76	[1.00, 3.09]	.05	1.19	[0.71, 2]	.52
Asian	1.58	[1.20, 2.07]	.00	1.02	[0.79, 1.3]	.90
Hispanic	1.43	[0.76, 2.69]	.26	1.09	[0.62, 1.91]	.77
Other race/ethnicity	1.18	[0.64, 2.18]	.59	1.38	[0.82, 2.34]	.23
**Job position (ref: optometrists)**^**a**^						
Ophthalmologists	0.84	[0.48, 1.47]	.54	0.50	[0.31, 0.82]	.01
Staff	1.41	[0.87, 2.3]	.17	1.12	[0.73, 1.73]	.60
Students	1.17	[0.78, 1.74]	.45	1.19	[0.84, 1.69]	.32
Childcare responsibility	0.81	[0.72, 0.92]	<.01	0.87	[0.79, 0.96]	.01
Daily new cases (per thousand)	1.97	[0.45, 8.73]	.37	1.40	[0.39, 5.06]	.61
Total cases (per thousand)	0.99	[0.97, 1.02]	.68	0.99	[0.97, 1.01]	.35
Patient interaction-weeks since 3/15/2020)	1.21	[0.99, 1.49]	.07	1.16	[0.97, 1.39]	.10
Patient interaction-days/week (past 2 weeks)	0.86	[0.78, 0.95]	<.01	0.95	[0.88, 1.04]	.26

**Notes:** ^a^ Gender, race, and job position were all dummy coded with the reference group noted in parentheses

## Discussions

Using validated questionnaires, this study focused on symptoms of depression, anxiety, and stress and revealed a high prevalence (38.4%) of probable depression and anxiety problems among eye care professionals and students. In addition, the linear and logistic regression analyses showed that being female, young, Black, or Asian predicted more mental health issues during the COVID-19 pandemic, while having greater proportions of childcare responsibilities at home, as well as more frequent interactions with patients protected against depression.

Torjesen reported a survey conducted by British Medical Association in May 2020 [26], which found that 41% of medical doctors were dealing with depression, anxiety, stress, burnout, emotional distress, or mental health condition worsened by work; 29% of respondents said these got worse during the pandemic [26]. Our finding that 38.4% of the surveyed eyecare professionals, staff, and students reported symptoms of depression, anxiety, or both during the COVID-19 pandemic is consistent with previous findings. However, future studies are needed to determine whether eye care professionals have worse mental health during the pandemic than other medical doctors.

Our finding that that female and younger eye care professional showed greater depression or anxiety symptoms during the COVID-19 pandemic is consistent with recent findings [3, 4, 27–30]. Li et al. showed that young physicians in China have higher mental stress [4], and Pieh et al. found that the COVID-19 pandemic seems particularly stressful for younger adults (< 35 years), and women [27]. In addition, Power et al. concluded that the psychosocial effects of COVID-19 disproportionately affect young people [28]. They reported that both immediate and longer-term factors through which young individuals were affected include social isolation, changes to the delivery of therapeutic services, and almost complete loss of all structured occupations (school, work, and training) [28]. Lai et al. reported female health care providers were more likely to experience stress and anxiety during the COVID-19 pandemic [3, 31]. Almeida et al. conducted a narrative review of published articles on women’s mental health and COVID-19 [30]. They concluded that women have a higher prevalence of risk factors known to intensify during a pandemic, including chronic environmental strain and stress [32], preexisting depressive and anxiety disorders [33], and domestic violence [34]. Thus, mental health interventions among eyecare professionals need to pay special attention to the younger and/or female sector of this population, which may include students and early-career eyecare professionals.

In addition, we found that both Black and Asian eye care professionals demonstrate greater depression symptoms and higher psychological stress than other race groups. Racial inequality and racism likely have contributed to these effects: Incidence rate and severity of COVID-19 are higher in the African American population, which could contribute to heightened stress and mental health issues among Black eye care professionals [35, 36]. Liu et al. showed that Asian young adults reported lower level of mental health symptoms than young adults of other racial background in the early phase of the COVID-19 pandemic [37]. However, the anti-Asian sentiment in America that has become associated with the origin of COVID-19 may have contributed to an increasing impact on Asian Americans as the pandemic progressed, and our survey may have picked up on this effect that was previously undetectable, since our data were collected in June–July 2020 while data in Liu et al. were collected in April–May 2020. More recently, Hahm et al. investigated the prevalence of COVID-19 related discrimination and reported that 68% of Asian and Asian American (A/AA) young adults reported that they or their family experienced COVID-19-related discrimination and approximately 15% of respondents reported verbal or physical assaults [38]. Thus, more attention needs to focus on the mental health issues in the Asian subpopulation among eyecare professionals during the COVID-19 pandemic.

The results on job category are mixed: While we found students to be more susceptible to depression and psychological stress than faculty and staff in the eye care profession in linear regressions, even when age is controlled for, this result did not appear in logistic regressions. The finding that ophthalmologists were at lower risk for anxiety only emerged in logistic regression but was only marginally significant in linear regression. Therefore, we recommend caution in interpreting these results regarding the association between job categories within eye professionals and mental health risk. There is one caveat: Given the consistent finding that younger age is a risk factor for mental health issues, students are still especially vulnerable to mental health issues due to their younger age.

The current research also identified two counter-intuitive effects: More frequent interactions with patients protected against depression symptoms and having a greater proportion of childcare responsibilities at home also protected against depression. Our initial predictions were the opposite. We predicted that childcare responsibilities would impose extra stress and challenge to the ophthalmic personnel during the COVID-19 pandemic based on previous research; for example, Almeida et al. proposed that parenting might be substantially more stressful during a pandemic because of the additional time required for caring for children and providing social support [30]. However, other research has also reported positive effects of family interactions and support on mental health [39–41]. During the particular social context of the COVID-19 pandemic, our findings show that eyecare workers did benefit from having a greater portion of the childcare responsibilities at home, potentially due to the beneficial effect of family interactions in caring for children. In terms of the finding that greater interactions with patients protected against depression symptoms, it is at odds with previous findings that work responsibilities could contribute to mental health problems, such as over-work and relationship with employers [42, 43]. On the other hand, during a time of social isolation caused by the COVID-19 pandemic and stay at home orders, the social benefits provided by interactions with patients may outweigh the potential negative impact of work-related stress. Indeed, our study showed that during the early phases of the COVID-19 pandemic, eyecare workers benefited from patient interactions even though these interactions were part of their work responsibilities.

During the COVID-19 pandemic, multiple efforts have already been put in place to reduce stress and manage mental health symptoms for health care workers, including: a hotline for health care worker [44], mental health support initiatives and lessons [45], social support [46], remote consultations [47], establishing principles of mental health care [48], implementation of tele-education [49], etc. The fact that 38% of eyecare professionals in the current study still demonstrate potential clinical depression and anxiety indicate that further monitoring and specific interventions on mental health for eye care practitioners are needed during the COVID-19 pandemic.

This study has a few limitations. First, this study might underestimate the impact of the COVID-19 pandemic on mental health because the study was conducted before the United States reached the peak of COVID-19. By the end of this study, July 8, 2020, total cases at USA exceeded 3,000,000 [20]. However, case number continued to increase to 4,000,000 by July 24, 2020 and over 5,000,000 by August 9, 2020 [20]. One might expect mental health to worsen with greater daily infections occurring in the United States. Our study group conducted another study to determine the longitudinal effect of COVID-19 pandemic on mental health symptoms and the effect of vaccination status on mental health in ophthalmic personnel [50, 51]. Another limitation is the potential selection bias in the sample. Because participants were self-selected into the study, those with even more severe mental health problems may not have participated the study. Alternatively, self-selection may have acted in the opposite direction, so that those who were less impacted by COVID-19 may not have felt motivated to respond to the survey. Finally, the survey study had a response rate of 25%, and recruited a convenient sample of participants through professional connetions, which may not provide great representation of eyecare professionals in general. Although high response rate in external surveys is difficult to achieve, with an average response rate about 10–15% [52], future research should strive to use random samples to achieve greater representativeness and generalizability.

In summary, this study is the first to examine mental health of eye care professionals in the United States during the COVID-19 pandemic, to the best of our knowledge. The findings of this study showed a high prevalence of mental health problems and revealed disparities in mental health among eye care personnel and students: Female, younger, Black and Asian populations are particularly vulnerable to these mental health issues. These findings shed important light on our understanding of how the COVID-19 pandemic impacts mental health among eye-care professionals, and particularly provides insights on the disproportionate impact among minority groups in this population. Based on these findings, we recommend that policies and strategies to improve mental health should be developed to help eyecare professionals and students cope with the negative impacts of the COVID-19 pandemic. In addition, we recommend that greater resources be allocated to developing and implementing effective mental health interventions that will particularly benefit female, younger, and racial minorities among eyecare professionals and students.

## Supplementary Information

**Additional file 1: Appendix.** Survey Questions.

## Data Availability

The datasets generated and/or analyzed during the current study are available in the. Open Science Framework repository at https://osf.io/6qfru/.

## Abbreviations

GAD-2: 2-item Generalized Anxiety Disorder scale (measuring anxiety disorder)
HIPPA: Health Insurance Portability and Accountability Act
IRB: Institutional Review Board
NPR: National Public Radio
OR: Odds Ratio
PHQ-2: 2-item Patient Health Questionnaire (measuring depression disorder)
PSS-4: 4-item Perceived Stress Scale
U.S.: United States of America
