# Supplementary material for: Risk factors for mental health symptoms during the COVID-19 pandemic in ophthalmic personnel and students in USA (& Canada): a cross-sectional survey study
Source: BMC Psychiatry. 2021 Oct 26;21:528. doi: 10.1186/s12888-021-03535-1 (PMC8546753; doi:10.1186/s12888-021-03535-1)
Supplement: Supplementary file 1 — Additional file 1: Appendix. Survey Questions. [file 12888_2021_3535_MOESM1_ESM.docx]

## **Appendix: Survey Questions**

1. How do you define your gender?

☐ Male

☐ Female

☐ Non-Binary

☐ Trans Male

☐ Trans Female

☐ Trans Non-Binary

☐ Identity Not Listed

☐ Decline to specify

1. How do you define your race?

☐ White/ Caucasian

☐ Black/ African American

☐ Asian

☐ American Indian/Alaska Native

☐ Native Hawaiian or Other Pacific Islander

☐ More than one race

☐ Decline to specify

1. How do you define your ethnicity?

☐ Hispanic/Latino

☐ Non-Hispanic/Latino

☐ Decline to specify

1. How do you define your stress level before COVID 19 in scale 1 to 5?

(1 means no stress and 5 is highest in stress level)?

☐ 1

☐ 2

☐ 3

☐ 4

☐ 5

1. How do you define your stress level over the last 2 weeks in scale 1 to 5?

(1 means no stress and 5 is highest in stress level)?

☐ 1

☐ 2

☐ 3

☐ 4

☐ 5

1. Do you have childcare responsibilities while working in the last 2 weeks?

☐ No, no childcare responsibilities

☐ Yes, <25% of childcare work is my responsibility

☐ Yes, 25 - 50% of childcare work is my responsibility

☐ Yes, 50 - 75% of childcare work is my responsibility

☐ Yes, >75% of childcare work is my responsibility

Over the last 2 weeks, how often have you been bothered by any of the following problems listed from questions 7 to 10

1. Little interest or pleasure in doing things

☐ Not at all

☐ Several days

☐ More than half the days

☐ Nearly every day

1. Feeling down, depressed, or hopeless

☐ Not at all

☐ Several days

☐ More than half the days

☐ Nearly every day

1. Feeling nervous, anxious, or on edge?

☐ Not at all

☐ Several days

☐ More than half the days

☐ Nearly every day

1. Not being able to stop or control worrying

☐ Not at all

☐ Several days

☐ More than half the days

☐ Nearly every day

1. How do you describe your current job/position?

☐ Ophthalmologist in private practice, group practice, or hospital

☐ Ophthalmologist in academic setting

☐ Optometrist in private, group, or corporate practice

☐ Optometry student (interact with patients)

☐ Optometry student (no interaction with patients)

☐ Staff in clinic (interact with patients)

☐ Staff not in clinic (no interaction with patients)

☐ Faculty in clinic (interaction with patients)

☐ Faculty not in clinic (no interaction with patients)

1. Which state have you been lived in the last two weeks?
2. From March 15^th^ until today, how many weeks have you been interacting with patients?

☐ 0 week

☐ 1 – < 4 weeks

☐ 4 - < 8 weeks

☐ > 8 weeks

1. How many days a week have you been interacting with patients during the last two weeks?

☐ No interact with patient

☐ 1 day

☐ 2 day

☐ 3 day

☐ 4 day

☐ 5 day or more

1. **In the last 2 weeks, how often have you felt that you were unable to control the important things in your life?**

☐ Never

☐ Almost never

☐ Sometimes

☐ Fairly often

☐ Very often

1. **In the last 2 weeks, how often have you felt confident about your ability to handle your personal problems?**

☐ Never

☐ Almost never

☐ Sometimes

☐ Fairly often

☐ Very often

1. What is your age?

☐ 20-29

☐ 30-39

☐ 40-49

☐ 50-59

☐ 60-69

☐ 70-79

☐ 80+

1. **In the last 2 weeks, how often have you felt that things were going your way?**

☐ Never

☐ Almost never

☐ Sometimes

☐ Fairly often

☐ Very often

1. **In the last 2 weeks, how often have you felt difficulties were piling up so high that you could not overcome them?**

☐ Never

☐ Almost never

☐ Sometimes

☐ Fairly often

☐ Very often

1. Have the following aspects of life during COVID 19 positively impacted your mental health? (Please check all that apply. If none applies, do not check any option)

☐ More free time

☐ More family time

☐ More sleep

☐ Being able to do things I couldn’t do before

☐ Less commute

☐ Being able to improve my academic/work performance

☐ Others (please fill in):________________________

1. Have the following aspects of life during COVID 19 negatively impacted your mental health? Please indicate the scale from 1 to 5 for each aspect (Please check all that apply).

|  | **1** | **2** | **3** | **4** | **5** |
| --- | --- | --- | --- | --- | --- |
|  | Not at all impacted | A little impacted | Moderately impacted | Much impacted | Overwhelmingly impacted |
| ☐ Worries about being infected with COVID 19 myself due to interactions with patients |  |  |  |  |  |
| ☐ Worries about transmitting COVID-19 to my family members due to my interactions with patients |  |  |  |  |  |
| ☐ Worries about being infected with COVID 19 myself in other aspects of life |  |  |  |  |  |
| ☐ Worries about family members/friends being infected with COVID-19 in other aspects of life |  |  |  |  |  |
| ☐ Worries about losing my job due to COVID-19 |  |  |  |  |  |
| ☐ Financial burden/ income reduction due to COVID-19 |  |  |  |  |  |
| ☐ Greater difficulty maintaining my academic/work performance compared to before COVID 19 |  |  |  |  |  |
| ☐ Greater difficulty handling house work or child care responsibilities compared to before COVID-19 |  |  |  |  |  |
| ☐ Social Isolation |  |  |  |  |  |
| ☐ Others (please fill in):________________________ |  |  |  |  |  |

1. Overall, how would you describe the impact of COVID 19 on your mental health?

☐ Overall, COVID 19 has a negative impact on my mental health

☐ Overall, COVID 19 has a positive impact on my mental health

☐ Overall, COVID 19 has no impact on my mental health

1. Which of the following have helped you maintain your mental health during the time of COVID-19? (please check all that apply)

☐ Exercise

☐ Outdoor activities

☐ Family time

☐ Social time

☐ Social media

☐ Work or study

☐ Others (please fill in):__________
